# An overview of Indian research in anxiety disorders

**DOI:** 10.4103/0019-5545.69234

**Published:** 2010-01

**Authors:** J. K. Trivedi, Pawan Kumar Gupta

**Affiliations:** Department of Psychiatry, C.S.M. Medical University, U.P. (erstwhile K.G. Medical University), Lucknow - 226 003, India

**Keywords:** Anxiety disorder, Indian studies and review, neurotic disorders

## Abstract

Anxiety is arguably an emotion that predates the evolution of man. Its ubiquity in humans, and its presence in a range of anxiety disorders, makes it an important clinical focus. Developments in nosology, epidemiology and psychobiology have led to significant advancement in our understanding of the anxiety disorders in recent years. Advances in pharmacotherapy and psychotherapy of these disorders have brought realistic hope for relief of symptoms and improvement in functioning to patients. Neurotic disorders are basically related to stress, reaction to stress (usually maladaptive) and individual proneness to anxiety. Interestingly, both stress and coping have a close association with socio-cultural factors. Culture can effect symptom presentation, explanation of the illness and help-seeking. Importance given to the symptoms and meaning assigned by the physician according to their cultural background also differs across culture. In this way culture can effect epidemiology, phenomenology as well as treatment outcome of psychiatric illness especially anxiety disorders. In this review an attempt has been made to discuss such differences, as well as to reflect the important areas in which Indian studies are lacking. An attempt has been made to include most Indian studies, especially those published in Indian Journal of Psychiatry.

## INTRODUCTION

Anxiety is arguably an emotion that predates the evolution of man. Its ubiquity in humans, and its presence in a range of anxiety disorders, makes it an important clinical focus. Developments in nosology, epidemiology and psychobiology have significantly advanced our understanding of the anxiety disorders in recent years. Advances in pharmacotherapy and psychotherapy of these disorders have brought realistic hope for relief of symptoms and improvement in functioning to patients.

## ANXIETY DISORDERS

The word anxiety is derived from the Latin “anxietas” (to choke, throttle, trouble, and upset) and encompasses behavioral, affective and cognitive responses to the perception of danger. Anxiety is a normal human emotion. In moderation, anxiety stimulates an anticipatory and adaptive response to challenging or stressful events. In excess, anxiety destabilizes the individual and dysfunctional state results. Anxiety is considered excessive or pathological when it arises in the absence of challenge or stress, when it is out of proportion to the challenge or stress in duration or severity, when it results in significant distress, and when it results in psychological, social, occupational, biological, and other impairment.

## CLASSIFICATION OF ANXIETY DISORDERS

The DSM-IV (American Psychiatric Association)[1] includes the following major categories of anxiety disorders: Panic disorder (with or without agoraphobia), agoraphobia without panic, social phobia (social anxiety disorder), specific phobia, generalized anxiety disorder (GAD), acute stress disorder, posttraumatic stress disorder, obsessive compulsive disorder, and anxiety disorder not otherwise specified. DSM-IV also lists anxiety occurring as an adjustment disorder, or secondary to substance abuse or a general medical condition. Finally, anxiety not amounting to a psychiatric diagnosis could be situational in normal persons, or a symptom of another psychiatric disorder.

## WHY THIS REVIEW OF ANXIETY DISORDER RESEARCH IN INDIA

Neurotic disorders are basically related to stress, reaction to stress (usually maladaptive) and individual proneness to anxiety. Interestingly, both stress and coping have close association with socio-cultural factors. Culture can affect symptom presentation, explanation of the illness and help-seeking. Importance given to the symptoms and meaning assigned by the physician according to their cultural background also differ across culture. In this way culture can affect epidemiology, phenomenology as well as treatment outcome of psychiatric illness especially anxiety disorders. In this review an attempt has been made to highlight on any such difference if there, as well as this review will also reflect the important areas, in which Indian studies are lacking.

This review will summarize most Indian studies pertaining to anxiety disorders published in Indian Journal of Psychiatry as well as found in other journals too.

## EPIDEMIOLOGICAL STUDIES

To the author’s knowledge there are three meta-analyses of Indian epidemiological studies of psychiatric disorders. A meta-analysis of 13 psychiatric epidemiological studies (Reddy and Chandrashekhara)[2] with a total sample size of 33,572 subjects who met the following criteria; door-to-door survey, all age groups included and prevalence rate for urban and rural being available, yielded an estimated prevalence rate of 20.7% (18.7-22.7) for all neurotic disorders, which was reported to be highest among all psychiatric disorders. The weighted prevalence rates of different anxiety disorders were 4.2% (Phobia), 5.8% (GAD), 3.1% (Obsession) and 4.5% (Hysteria). Panic disorder was not included in this meta-analysis and the reason for this is surprisingly not discussed. This meta-analysis also reported that prevalence rates of all neurotic disorders except hysteria (5.0% vs. 3.4%, *P* < 0.5) were significantly higher (35.7% vs. 13.9%, *P* < 0.01) in urban communities than rural, and all neurotic disorders were significantly high among females (32.2% vs. 9.7%, *P* < 0.01). Though meta-analysis has its own limitations, this was the first attempt to analyze the epidemiological studies.

It has been seen that rural epidemiological studies are more difficult to conduct as compared to urban ones, due to ignorance, stigma and lack of resources. Disorders like obsessive compulsive disorder often go unaccounted due to ignorance and attribution of such issues to personality factors.[3] This can be a possible explanation for higher prevalence of anxiety disorders in urban areas than to the same in rural areas. Disorders like hysteria are accounted in a more reliable manner and are significantly more common in rural communities because of visible manifestation of the disease (Reddy and Chandrashekhara).[2]

Ganguli[4] analyzed 15 epidemiological studies on psychiatric morbidity in India. In this meta-analysis prevalence rate (in per thousands) of anxiety neurosis was reported to be 16.5 with a rural urban ratio of 100:106 and that of hysteria was 3.3 with a rural urban ratio of 100:44. These findings of meta-analysis were consistent with that of reported in meta-analysis by Reddy and Chandrashekhara.[2] Except hysteria, the prevalence rates of various anxiety disorders included in the anxiety neurosis were not separately assessed, thus leaving us blindfold in the overall affliction of the population from these individual disorders.

Madhav,[5] in an analysis of 10 Indian studies on psychiatric morbidity, concluded that prevalence rates for anxiety neurosis and hysteria were 18.5 and 4.1 per 1000 population respectively. The common feature of the two meta-analyses described above was that they included studies which were conducted in three steps or phases;

1. Delineation of the sample and initial contact with subjects including collection of background demographic data.
2. Identification of suspected cases, usually on the basis of interviews and questionnaire by non-psychiatric personnel like social workers and sometimes by psychological tests. Physical examination of suspected cases by medical personnel was part of this phase.
3. Psychiatric examination and clinical diagnosis and classification of suspected cases were the third stage.

### Epidemiology of anxiety disorders in elderly

Epidemiological data of anxiety disorder in special population like pediatric and elderly are scant. To the best of the author’s knowledge, one population-based study on geriatric population was reported by Tiwari and Srivastava.[6] These authors identified 488 elderly subjects in a rural region of Uttar Pradesh. Nearly 9% of the subjects were diagnosed with ICD-9 (World Health Organization)[7] anxiety neurosis. These data may contain unknown biases because over 42% of the geriatric population was assigned a psychiatric diagnosis; in contrast, less than 4% of non-geriatric subjects had an ICD-9 psychiatric diagnosis.

### Epidemiology of anxiety disorders in pediatric population

A prevalence rate of psychiatric morbidity among pediatric population has been reported since very early (Sethi *et al*.)[8,9] but these studies did not report prevalence rates of anxiety disorders separately. In one of the earliest known report on neurotic disorders, Nagaraja[10] observed childhood neuroses in 9.7% of out-patient population and 9.3% of inpatients over a period of seven years in Hyderabad with a male to female ratio of 1:2. Manchanda *et al*.[11] found neurotic behavior in 27.3% children admitted for physical ailments. In children seen at the Child Guidance Clinic of the Madras Government General Hospital during the year 1964-1966, Raju *et al*.[12] found that 22 of the 592 children were neurotics and 16 were hysterical. Later, in an urban survey of 109 families for psychiatric morbidity in children below 12 years, Lal and Sethi[13] reported emotional disturbance in 55% families and 35.4% of the total children surveyed. Neurotic disorders were found in 11.0% of the total sample, but the clinical states mentioned therein were extremely varied and did not follow any classificatory scheme.

In an another epidemiological study conducted by Manchanda and Manchanda,[14] a total of 19 children (up to 12 years) from the Pediatric inpatient and Child Guidance Clinic (CGC) were diagnosed to be suffering from a neurotic disorder during a period of 11 months. Incidence of neuroses was 1.1% among pediatric inpatients and 8.2% in CGC. The incidence was higher in the females. 73.5% of children were in the age range of 10-12 years. None of them were below six years. Hysteria was the commonest diagnostic group (71.4%) in the present study. Therefore, it is likely that the findings observed for neurotic disorders in general, are more characteristic of hysteria. Other disorders in order of frequency were anxiety (16.3%) depression (6.1%) and phobia (4.1%). Obsessive compulsive neurosis was observed in one case only.

In another epidemiological study of possession syndrome (Venkataramaiah *et al*.)[15] conducted in West Karnataka reported high prevalence rates of 51% in age group <15 years and 28% in age group 15-25 years (n = 718). In this study a house to house survey was conducted for a population of 1158 in west Karnataka to determine the prevalence of possession syndrome and to study people’s attitude towards the same. One year period prevalence was found to be 3.7%.

Recently a community-based study was conducted by ICMR in Lucknow (ICMR,)[16] and Bangalore (Srinath *et al*.)[17] in children and adolescent age 0-6 years. The prevalence rates of various anxiety disorders, reported in the study are shown in the Table 1.

**Table 1:** Prevalence of anxiety disorders in pediatric population

Disease as per ICD-9	In Lucknow center (n = 2325) (%)	At Bangalore center (n = 1578) (%)
Social phobia	0.19	0.19
SAD	0.09	0.2
GAD	0.14	0.3
Simple phobia	1.98	2.9
Agoraphobia	0.05	0.1
Panic	0.05	0.1
OCD	0.09	0.1
Conversion disorders	0.17	Not reported

## CONCLUSION

Most of the epidemiological studies done in India neglected anxiety disorders. The use of poor sensitive screening instruments, single informant and systematic under- reporting has added to the discrepancy in the prevalence rate. The prevalence of mental disorders reported in epidemiological surveys can be considered lower estimates rather than accurate reflections of the true prevalence in the population (Math *et al*.).[3]

Most of the Indian epidemiological studies analyzed here have surveyed a population less than 6000. This raises a query whether the findings can be generalized to even one State of India. Mental health care priorities need to be shifted from psychotic disorders to common mental disorders like depression, anxiety disorders, somatoform disorder, etc., which are also associated with high disability in all measures (Patel *et al*.).[18]

## STUDIES RELATED TO PHENOMENOLOGY, CO-MORBIDITY AND OTHER CLINICAL VARIABLES

### Studies on hysteria, conversion or dissociative disorders [Table 2]

In children and adolescents, review of literature shows that common disorders seen are dissociative convulsions and stupor.[26–29] Symptoms of motor weakness, amnesia, and aphonia are less commonly seen. The common stressors reported were academic difficulties, family problems, peer problems, sibling rivalry and at times difficult situations like marriage etc.[30,26,31] Common co-morbidities reported were depressive disorder, anxiety disorders, adjustment disorders, oppositional defiant disorder and specific developmental disorder of scholastic skills.[28] Differential diagnoses could be epilepsy, involuntary movement disorders like chorea, dystonias, syncopal attacks, panic attacks and other neurological disorders. Good outcome of dissociative disorders have been reported.[26,28,29] Early diagnosis, presence of a psychosocial stressor and appropriate intervention has been associated with good out come in these patients.

**Table 2:** Studies on hysteria, conversion or dissociative disorders

Study	Sample	Method	Results and conclusion
Singh[19] A Clinical-psychological study of ‘Hysteria’	Fifty consecutive patients of both sexes were studied between the age ranges of 15-45 years	Detailed diagnostic Evaluation and assessment of personality by MPI.	- Hysterics comprise 8.3% of the clinic population.
			- Male female ratio:—11:39 (22%)
			- Most common symptoms were fainting attacks (25) convulsive fits (15) headache (15) abdominal pain (9) menstrual difficulties (7) and sinking sensation (6)
			- There are symptoms of dissociative reaction and conversion reaction (21:18) with some cases classed as mixed reaction and one case of hysterical psychosis.
			- Most frequent personality being the passive-aggressive and hysterical personality.
Bagadia *et al*.[20] Hysteria-A prospective study of demographic factors of 192 cases	192 diagnosed case of hysteria, out of 2926 attendees of OPD	Detailed psychiatric and physical evaluation	- Fifty six per cent of the cases belong to the age group of 16 to 25 years.
			- Occurrence of hysteria is significantly higher in females than males (78:22).
			- Occurrence was higher in unmarried males and married females.
			- Illness was more common in persons with lower education.
			- Unemployed males were significantly more (19%).
			- Occurrence was significantly high (26%) in those who were staying in Mumbai for less than one year.
Vyas *et al*.[21] A study of hysteria—an analysis of 304 patients		304 patients of hysteria were studied and they were analyzed under different demographic factors.	- High occurrence was seen in 16-25 years age group and the occurrence of hysteria was significantly high in females. More numbers of patients came from poor, low income families and from joint family system. There is a definite shift in the pattern of hysterical manifestations.
Subramanian *et al*.[22] A clinical study of 276 patients diagnosed as suffering from hysteria			- The peak age of onset was 10-20 years. The majority were married. 75% of them had conversion symptoms, 20.3% had dissociative states, and 4.7% had both features. 52.5% showed possible precipitating factors. 66.0% had features of extraversion in their personality make up. 14.1% showed evidence of parental deprivation. There was over-representation of the early born.
			- Somatic symptoms (aches and pains) were the most common mode of presentation. The other common clinical manifestations were fainting attacks, fits, vomiting, involuntary movements and paralysis of limbs. Only 93 patients could be contacted for the final follow-up. Among these, 28 recovered completely; 50 were improved; two became worse and two died.
Wig *et al*.[23] A follow up study of hysteria The present study undertook to examine the outcome of a group of cases who were diagnosed as hysteria, six or more years ago in a general hospital psychiatric unit and correlate various clinical factors with good or bad outcome	N = 57	Of the 81 cases selected for the study, 57 (67%) could be located and followed up after a gap of six to eight years	- Majority of the cases (74%) had either no symptoms or symptoms less than before at the time of the follow-up.
			- In only 3 cases, there was evidence of an underlying organic illness which seemed to have been missed at the initial assessment.
Bhargawa *et al*.[24] Study of illness behaviour in hysterical patients	N = 30	Patients diagnosed with conversion disorder and somatization disorder on DSM-IV were investigated using Illness Behavior Questionnaire (IBQ) and Eysenck Personality Inventory (EPI)	- The patients differed with the controls on all the 7 factors of illness dimensions. They scored higher on neuroticism and low on extroversion.
			- Understanding of the patterns of illness behavior can be utilized in the long term psychological management of the patients, where by tackling such factors as affective inhibition, denial of life problems, phobic concern about illness, etc, may facilitate better expression of emotional distress and amelioration of hysterical symptoms.
Deka *et al*.[25] A study of clinical correlates and socio-demographic profile on conversion disorders	N = 40 (inpatients) Subjects of both sexes of age >6 years fulfilling the diagnostic criteria for conversion disorders (ICD-10)	Patients were assessed for socio-demographic factors based on semi structured proforma	- Conversion disorder is more common in young adults (57.5%), females (92.5%), and among students belonging to nuclear families of lower socioeconomic status. Majority of the patients had obvious precipitating factors, of which family-related (40%) and school-related (30%) problem accounted for the major types motor symptoms were the predominant presentation (87.5%) with pseudo seizure being the commonest.

### Panic disorders

The phenomenology of panic disorder has been studied widely in the West but rarely in India. Srinivasan and Neerakal[32] studied 94 panic patients attending the OPD of psychiatry department. This study has shown considerable co-morbidity of major depression (according to DSM-IV criteria) in 43 patients (45.7%) with panic attacks. Majority (i.e. 69.8%) of the subjects with panic attacks had co-morbid primary depression and only 30.2% had secondary depression. More so, there was a greater prevalence of concurrent generalized anxiety disorder in panic patients with depression (both primary and secondary) as compared to panic patients without depression. Authors of this study have mentioned that their findings are in alignment with those of Western studies. Cross-sectional design of this study was its major limitation. A longitudinal study would have been more clearly identified the relationship between panic disorder and depression.

#### Social anxiety disorders

Social Anxiety Disorder (SAD) is a chronic, disabling and treatable disorder with common onset in adolescence. There is only one study conducted by Mehtalia and Vanker[33] to find out frequency, demographic and phenomenological characteristics of SAD, family related risk factors, academic impairment and co morbidity of depression among adolescents. A total of 421 adolescents in one high-school were screened for SAD and depression and associated factors with academic impairment. 54 (12.8%) had SAD. The most common manifestation of SAD was avoiding giving speeches. SAD was equally common among both genders, was associated with difficulty in coping with studies, concern about weight, having less friends, lack of intimacy with parents, and being treated differently from siblings. This study concluded that SAD is a common adolescent disorder, with major depression as co-morbidity and associated with impairment in academic functioning. All adolescents, especially with depression consulting medical professionals, should be interviewed for SAD and treated. The findings of this study are based on only one stage screening. The findings need to be replicated in further two-stage study employing structured clinical interview for more valid conclusions. Although this study has explored the most common co-morbidity i.e. major depression, other anxiety disorders and relationship with avoidant personality disorder has not been explored. Future studies on this aspect are also needed.

#### Post traumatic stress disorders

PTSD has a global significance, and its impact in countries that have been experiencing repeated disaster and social unrest for many years could be a large public health problem. Despite having a global significance, its prevalence has been best studied in industrialized societies only particularly in USA.[34] It is unclear whether US data can be generalized to other developed countries.[35] The situation is almost certainly quite different in the developing countries where large proportion of population have been exposed either directly or indirectly to terror attacks, torture, sexual assault, and forced relocation.[36–38] Estimates of the prevalence of PTSD in the general population of the countries other than America are lacking.[39] There are no published or detailed studies available so far from India except a few national and international conference presentations.[40–48] Published data in this area is dismally minimal and there is need for further research. Following are the studies related to PTSD, which have been published in recent years [Table 3].

**Table 3:** Studies related to PTSD

Sharan *et al*.[49]	Reported a 59% prevalence of psychiatric disorders in the adults following Marathwada earth-quake (23% had posttraumatic stress disorder (PTSD) and 21% had depression).
Gautam *et al*.[50]	Study on victims (n = 31) of bomb blast in a bus by terrorist activity reported that after 2 weeks of trauma the most common psychiatric diagnosis was PTSD (12.9%) followed by depression (9.6%) and dissociative amnesia (6.4%). Two (6.45%) subjects with PTSD also had co-morbid depressive episode. In this study, phenomenology of subjects with PTSD was not described separately. It was cross sectional study and various vulnerability factors associated with PTSD have not been studied.
Kar *et al*.[51]	Study on Mental Health Consequences of the Trauma of Super- Cyclone 1999 in Orissa reported prevalence rate of Posttraumatic stress disorder was 44.3%, after anxiety disorders (57.5%) and depression (52.7%).
	In this study, unemployment, death in family, seeing family member being washed away, seeing dead body, fear of death were associated with PTSD. Adolescents and elderly persons, death in the family, fear of imminent death during the event, hopelessness, increased stress before disaster and past psychiatric history were associated with adverse psychological sequelae.

#### Obsessive-compulsive disorders

The very first paper published in Indian Journal of Psychiatry, in 1951, was authored by Dr. P.K. Ray, titled “Common Obsession –compulsive symptoms in India”.[52] In this paper an attempt was made to relate abnormal mental phenomenon in India to the cultural and religious background of Hinduism as found in Bengal. But there was no attempt made at psychological and psycho-analytic interpretation of these symptoms. Since then there are a few studies related to phenomenology and various clinical variables have been published from India.

Akthar *et al*.[53,54] tried to delineate obsessions and compulsions based on form and content from a phenomenological view. They identified five types of obsessions: Doubts, obsessive thinking, fear, impulses and images. In compulsions, they identified two types, yielding and controlling compulsions. They identified six varieties of thought content: Dirt and contamination, aggression, inanimate-impersonal, sex, religion and miscellaneous. They opined that form is affected by intrinsic factors and content by extrinsic factors.

Khanna *et al*.[55] tried to establish a phenomenological system of classification for various phenomena observed in OCD, using a classificatory system for obsessions (Khanna and Channabasavanna),[56] and compulsions (Khanna and Channabasavanna),[57] which had high inter-rater reliability (Khanna *et al*.),[58] T hey derived 6 forms and 10 contents of obsessions and four forms and six contents of compulsions. These variables were used for cluster analysis. Seven clusters emerged, of which four were considered important. (1) Checking, (2) Washing, (3) Past, (4) Embarrassing behavior. Though washing and checking constituted two largest pure clusters, there was significant overlap, which is due to their frequent co-occurrence. These studies had their own methodological limitations. There are two more recent studies regarding the phenomenology and course of OCD, published in the IJP which has been tabulated below [Table 4].

**Table 4:** Recent Studies on OCD

Study and author	Sample and methodology	Results and conclusion
Girishchandra and Khanna[59]	A total of 202 consecutive subjects with OCD were evaluated using the Yale Brown Obsessive Compulsive Scale- Symptom Check List and Scale for Assessment of form and content. The data was subjected to Factor analysis with varimax rotation	The results suggest that there are factors which are broadly common to the two scales. The main factors which emerged were washers, checkers, hoarding and two pure obsessional factors. The cross-cultural validity of these factors has been established for an Indian population
Phenomenology of obsessive compulsive disorder: A factor analytic approach	The study is one of the few prospectively conducted studies, with larger sample size, was attempted to validate the classification with gratifying results.	The course of illness was found to be similar in both the subtypes
Math *et al*.[60]	This study compared the 5-6 years course of the “predominantly obsessive” (n = 38) with that of the “mixed” subtype (n = 39). ICD-10 and Y-BOCS were used for diagnosis and severity assessment respectively	Although the study has small sample and retrospective in design, it has an implication regarding the validity of subtypes of OCD. Future studies of subtypes of OCD are required in larger sample with focus on neurobiologic and genetic parameters

## BIOCHEMICAL STUDIES

Understanding the biology of various psychiatric disorders has taken the front seat in psychiatric research. Indian studies particularly on this area of anxiety disorders are very less [Table 5].

**Table 5:** Biochemical studies on anxiety disorders

Author and study	Sample	Methodology	Results and conclusions
Rao *et al*.,[61] A study of spontaneous functional hypoglycaemia in relation to anxiety	Series of 9 patients out of 42 patients of anxiety were included who have developed with predominant symptoms of anxiety related to food intervals	Complete psychiatric (history and MSE) and physical evaluation, routine urine examination, and GGT was done using the Somogyi-Nelson method. In this the last specimen of blood was taken at the end of 3 hours.	Two patients did not improve at all, 4 patients improved significantly and tranquillizers discontinued. Three patients showed some improvement and they were able to adjust, with weekly supportive therapy The spontaneous hypoglycemia of functional origin is not so uncommon. it is worth-while to do a G.T.T. in all cases of anxiety reaction arising in relation of food
Singh *et al*.[62] Response to pineal gland in clinical cases of psychological stress	Fifty patients of thyrotoxicosis (25) and anxiety neurosis (25) suffering from psychological stress	Biochemical estimation of melatonin was done in all these cases prior to treatment and 3 months after appropriate treatment.	A significant increase in the level of melatonin was observed in thyrotoxic and anxiety neurotic cases. In all these cases, after therapy the level of melatonin was found to be within the normal limits. These observations confirm the finding that there is a pineal response to psychic stress.
Mishra *et al*.[63]	36 patients of anxiety neurosis diagnosed as per Feighner’s diagnostic criteria (Feighner *et al*.),[64] attending the OPD were included with 24 control subjects.	The patients were assessed on Hamilton anxiety scale	Patients of anxiety neurosis showed hyper triglyceridemia and increased VLDL-cholesterol concentration with reduction in esterified cholesterol level. First two findings reflect that anxiety neurotics are at a greater risk for the development of atherosclerosis and its cardiovascular complications and that TGs, VLDL, cholesterol and esterified cholesterol are perhaps the biochemical variables mediating the cardiovascular psychosomatic response. The assumptions are tentative, and regretfully have never been studied again.

## STUDIES ON COGNITION IN ANXIETY DISORDERS

Only a few studies have assessed neuro-cognitive domains in anxiety disorders. A study by Tarafder *et al*.[65] on 20 patients of OCD found impairment of executive functions when assessed on WCST. Another study by Trivedi *et al*.[66] on 30 patients of OCD found that the patients of OCD performed significantly worse on cognitive measures (evaluated on WCST, CPT and SWMT) than healthy controls.

## STUDIES ON LIFE EVENTS AFFECTING ANXIETY DISORDERS

Bhatti and Channabasavanna[67] studied neurosis through stressful life events, personality dimensions, family interactional patterns and other sociological variables. They studied 60 neurotics and 60 controls, 92% respondents had stress in more than one area like work, education, family etc in the experimental group. The mean number of stressful life events experienced by neurotics over a period of one year was around 5, which is much higher than the normal population. Only 40% respondents in the control group had stress in just one area.

Sharma and Ram[68] carried out a study on 84 patients of anxiety neurosis and 47 controls. On assessing the life events during life time and six months prior to the onset of illness by an open ended interview, using a scale suited for Indian population, frequency and stress scores experienced by patients and by controls was observed. It was observed that a variety of events were significantly more frequent in the patient group. Events related to personal, social, sexual, educational, occupational and financial areas were observed significantly more in patients during life time and six months prior to the onset of illness. Four events, namely, suspension from job, theft or robbery, broken engagement or love affair and conflict over dowry were found to be significantly more in patients during lifetime. Four other life events such as major purchase or construction of house, failure in exam, appearing for interview and getting engaged or married were found to be significantly more in patients during the six months prior to the onset of the illness. Thus patients experience a variety of life events often more often than the controls.

## STUDIES BASED ON TREATMENT AND MANAGEMENT OF ANXIETY DISORDERs

Studies based on treatment of anxiety states or disorders are being published in Indian Journal of Psychiatry since 1959. The initial few published studies have been - Effect on anxiety states of carbon dioxide Jetley,[69] and Guaiacol glycerol ether (Mehta *et al*.),[70] Trial of haloperidol in anxiety states (Jairam and Ram),[71] double blind placebo-controlled trial of Trioxazine (Katira and Iyer),[72] clinical trial of Pimozid in anxiety (Ramchandran and Menon),[73] and study of prochlorperazine in anxiety disorders (Nigam *et al*.)[74]

The first Indian study on the effect of Benzodiazepines in anxiety disorders was conducted by Master and Kajaria.[75] This double-blind study was done on 60 outpatients to compare the efficacy of Lorazepam and Diazepam in anxiety neurosis. It concluded that both Lorazepam and Diazepam are effective anxiolytics but a clinically satisfactory response occurs earlier with Lorazepam. Effect of Clobazam, a nonbenzodiazepine anxiolytic was studied by Singh *et al*.[76] and later compared with the Diazepam (Channabasavanna and Pereira).[77]

Khanna *et al*.[78] studied 12 subjects with a diagnosis of OCD who had not shown response to Amitryptiline and Imipramine/behavior therapy those subjects underwent a cross over double blind trial with Clomipramine and Nortryptiline. Subjects who had earlier not shown response to the other drugs did not show response to Clomipramine. This study provides tentative evidence that an adequate trial of Imipramine and Amitryptiline should be given in all cases of OCD, and that if subjects do not respond to these two drugs, it is unlikely that they will show response to Clomipramine.

Shah *et al*.[79] conducted a controlled double blind trial of Buspirone and Diazepam in generalized anxiety disorder. Patients in both groups showed improvement on Hamilton Anxiety Scale. However, in the Buspirone group, improvement was seen in cardiovascular, somatic autonomic, anxious and mood symptoms; while in the diazepam group, improvement was noticeable in anxious mood, tension, insomnia, cognitive symptoms, somatic and cardiovascular symptoms. The mean total daily dosage required by the patient in the Buspirone group was 36.56 mg/ day, which was more than reported elsewhere.[80,81] More patients in the Buspirone group dropped out midway in the trial compared to Diazepam group. The lag time of anxiolytic efficacy of Buspirone is longer and thus motivation for compliance is necessary.

Shah *et al*.[82] evaluated Alprazolam and Diazepam in GAD, diagnosed by DSM III criteria in a double blind multicentric study. Weekly evaluations were systematically carried out for a period of four weeks; 148 patients (79%) completed the trial. Results showed that Alprazolam was as effective as diazepam as an anxiolytic. Drowsiness was less often reported with Alprazolam. This was the short follow-up study; the efficacy of Alprazolam in long term use needs to be evaluated.

Sahasi *et al*.[83] ascertained the effectiveness of different relaxation techniques in the management of anxiety. Earlier study by Sahasi *et al*.,[84] found significant improvement among patients undergoing yoga therapy compared to those taking minor tranquilizer (Diazepam). In the 1991 study, psychological and self report data were obtained from the participants practicing progressive relaxation and yogic techniques of relaxation. Both techniques generated positive expectancies and produced a decrease in a variety of self-reported symptoms. Yogic techniques produced greater motivation to practice than progressive relaxation. The follow-up rate was much better among the yoga group than those who were doing progressive relaxation. Yogic techniques are more readily acceptable by our population. Following the yogic way of life probably acts as a psycho prophylactic against anxiety.

Vahia *et al*.[85] conducted a study to compare the efficacy of meditation with that of Imipramine and Chlordiazepoxide in the treatment of GAD, diagnosed as per DSM III criteria. At the end of five weeks, meditation was found to be as effective as pharmacotherapy in controlling symptoms of anxiety. It was superior in altering trait anxiety. Meditation is an easy to learn and effective therapy. It has a distinct advantage over pharmacotherapy in that it does not have the associated problems of habit formation, withdrawal effects, over dosage or other undesirable effects.

Andrade *et al*.[86] conducted a double-blind controlled evaluation of the efficacy and adverse effect profile of sustained release Alprazolam. Disadvantage of Alprazolam is that its anxiolytic efficacy wears off much earlier than the drop in its blood levels. Therefore, thrice or even 4 times daily dosing may be necessary, despite which inter-dose anxiety is some times a clinical problem. In patients with panic disorder, sustained release Alprazolam was found to be as effective as conventional Alprazolam, the SR formation was also well tolerated (Schweizer *et al*.).[87] India is probably the only country in which a sustained release preparation of Alprazolam is commercially available. 40 patients with GAD, as per DSMIV diagnosis and stabilized on Alprazolam therapy were randomized to receive first the same dose of either conventional or sustained release Alprazolam for two weeks, followed by the other formulation of Alprazolam in an identical dose for a further two weeks. No efficacy difference was observed between the two forms of Alprazolam. Once-daily SR formulation is as effective as the conventional form of the drug. It’s use in drug naive patients and examining the long-term efficacy, compliance and withdrawal in naturalistic studies would be essential.

## CONCLUSION

Status of anxiety disorder research from India in relation to epidemiology, phenomenology, course, outcome and management are lacking. Research areas like family studies, genetics, and neurobiology are not touched adequately. Most of the studies have tried to replicate the findings from the West. Despite rapid advancement in the field of psychopharmacology, the researches in the field of anti-anxiety and antidepressant drugs are dismally low from India. Furthermore research is lacking in the areas of non-pharmacological management like relaxation therapies, yoga, other meditation techniques and psychotherapies despite India being the birth place of many such techniques. Most of the research is done by tertiary centers involving limited sample which may not provide the real picture.
